# Supplementary material for: Factors associated with active syphilis among men and women aged 15 years and older in the Zimbabwe Population-based HIV Impact Assessment (2015–2016)
Source: PLoS One. 2022 Mar 17;17(3):e0261057. doi: 10.1371/journal.pone.0261057 (PMC8929562; doi:10.1371/journal.pone.0261057)
Supplement: Attachment — Submitted filename: Response to PLoS One January 2021.docx [file pone.0261057.s001.docx]

Dear Editor-

Thank you for pre-reviewing our manuscript titled, *Factors associated with active syphilis infection among men and women aged 15 year and older in the Zimbabwe Population-based HIV Impact Assessment (2015-2016)*. During your pre-review you noted a similarity between our paper and a manuscript published in PLoS One in July 2020 from Solomon et al also focused on syphilis and HIV co-infection in Zambia. You correctly note that several of the authors of this manuscript and ours are the same. The Population-based HIV Impact Assessments (PHIA) are a collection of centrally funded, but independent national HIV surveys. As such, methods are shared between countries, but the objectives of each assessment vary based on the nature of the HIV epidemic in the respective country. Thus, you’ll see similarities in authorship lists pertaining to the individuals who helped develop the laboratory and sampling methods for all the PHIAs. Zimbabwe and Zambia share a border, but their HIV epidemics differ vastly. We believe the differences between our HIV epidemics, and how comorbidities on a population level differ, warrant investigation and description. We have cited the work of Solomon et al from last year, and we are pleased to resubmit the manuscript for your consideration.

Sincerely,

John H. Rogers, PhD, MPH

Associate Director for Science

U.S. Centers for Disease Control and Prevention, Zimbabwe
