# Supplementary material for: Factors associated with active syphilis among men and women aged 15 years and older in the Zimbabwe Population-based HIV Impact Assessment (2015–2016)
Source: PLoS One. 2022 Mar 17;17(3):e0261057. doi: 10.1371/journal.pone.0261057 (PMC8929562; doi:10.1371/journal.pone.0261057)
Supplement: Attachment — Submitted filename: Response to Reviewers.docx [file pone.0261057.s002.docx]

Dear Editor-

Thank you for reviewing our manuscript titled, *Factors associated with active syphilis infection among men and women aged 15 year and older in the Zimbabwe Population-based HIV Impact Assessment (2015-2016)*. Below please find a point-by-point response to your review. We look forward to receiving any additional feedback and/or publication in PLOS ONE.

Unsolicited change:

Authorship order adjusted to reflect further effort by some authors. If allowable, Leala Ruangtragool and Rachel Silver should be credited as co-first authors having contributed equally to the manuscript. Likewise, Beth Tippett Barr and John H. Rogers should be credited as co-final authors and listed in the order now presented. Please let us know if this is acceptable.

Reviewer #1:

1. While generally well-written, the paper is overly long. A word count is not presented, but it appears to be well over the 3,000 word limit that is generally accepted for communications like this. Specifically, the Introduction does not need an extensive description of syphilis morbidity and the Results section is basically a verbatim recap of what’s already in the Tables and can be reduced to some of the highlights that are subsequently considered in the Discussion section. In that context, I have often wondered what the use is of repeating the 95% confidence intervals in the text, which, in my mind just impedes the narrative.

*Respectfully, PLOS ONE style requirements allow for manuscripts of any length. The body of the manuscript is approximately 3,300 words, which when considering the inclusion of measures of association in the body of the Results, is quite close to the referenced 3,000 word limit. The inclusion of 95% confidence intervals is a stylistic preference. We have elected to keep them in the body of the manuscript in order to provide the necessary statistical context of reported measures of association. Doing so keeps the reader from having to flip back and forth between the text and the tables and, from my perspective, aids readability. If the reviewer feels strongly that the introductory paragraph describing the phases of syphilis should be removed, then we are open to that suggestion, but its inclusion is intentional to set the stage for the rest of the manuscript by understanding the morbidity of syphilis.*

2. The Methods section, though, is short on details. Specifically, is suggests that HIV and syphilis testing were both done in the household settings and that results were given in that setting as well. However, there is no detail on what happened to study participants with positive results. Were they referred for clinical services? Was there follow up that treatment was assured? After all, some of these patients had active syphilis (and some may have had newly diagnosed HIV) with important consequences both clinically and epidemiologically for the prevention of ongoing transmission.

*Addressed in methods.*

3. The study employs Chembio’s Dual-Path Platform Syphilis Screen & Confirm test and defines “active syphilis” as positive results for both the treponemal and non-treponemal components of the test. However, there is no discussion on the limitations of this test, specifically the low sensitivity for both components in persons with low-titer non-treponemal test results by RPR (see for example: http://dx.doi.org/10.1136/sextrans-2018-053722). It can be argued that, from a clinical perspective it is particularly important to identify those syphilis patients with high titer non-treponemal test results as they have active disease (see for example: https://www.ncbi.nlm.nih.gov/pmc/articles/pmid/31008842/). However, from an epidemiological point of view (i.e., measuring prevalence and incidence), the sensitivity issue is more problematic, especially when comparing data over sequential surveys that may have used different technologies. At the least, this should be discussed as a limitation.

*Thank you for this comment. The manufacturer’s website claims 96.5% sensitivity for the treponemal component and 99.7% sensitivity for the non-treponemal component (for RPR titer >= 1:8). Two published evaluations also demonstrate good performance of the trep line compared to TPPA and the non-trep line compared to RPR/TRUST at titers >= 1:4 but report lower sensitivities in the non-trep line at titers of 1:2 or less (*[*https://www.ncbi.nlm.nih.gov/pmc/articles/PMC3657488/*](https://www.ncbi.nlm.nih.gov/pmc/articles/PMC3657488/) *and* [*https://www.ncbi.nlm.nih.gov/pmc/articles/PMC3008492/pdf/0624-10.pdf*](https://www.ncbi.nlm.nih.gov/pmc/articles/PMC3008492/pdf/0624-10.pdf)*). We could state this reported lower sensitivity of the non-trep line of the DPP Screen and Confirm test in specimens with a titer of 1:2 or less as a limitation of the survey methods, but question if it is necessary given the cited length of the manuscript and that a referenced work in PLOS ONE using the same methods (see* [*https://www.ncbi.nlm.nih.gov/pmc/articles/PMC7380641/*](https://www.ncbi.nlm.nih.gov/pmc/articles/PMC7380641/)*) did not cite this limitation.*

4. As a minor point, I take issue with the term “syphilis infection”, as an infection is not caused by its outcome. Similarly, we say: “gonococcal infection” and not “gonorrhea infection”. Therefore, keep it simply to “syphilis”.

*Addressed per recommendation throughout the manuscript.*

Reviewer #2:

1. Add findings related to overall burden of active syphilis by gender in results section of abstract. Authors highlighted that PHIA findings would also be beneficial for addressing the bias in syphilis estimates using ANC data and without highlighting burden of syphilis by gender, the findings related to association only is less important.

*Thank you for your comment. The prevalence of active syphilis by gender is presented in the first paragraph of the Results.*

2. Add separate section in methods to specify outcome of interest (outcome variables) so that it would be easy to follow for readers.

*The second paragraph of the statistical analyses section describes both the independent variables of interest and the dependent, or “outcome”, variable, which is active syphilis.*

3. Is it possible to present findings related to treatment status among pregnant women diagnosed with active syphilis? Also highlight prevalence of untreated syphilis by gender (is there any questions that asked ‘whether they seek any STI services in past etc)?

*Thank you for this comment. Active syphilis does not differ by pregnancy status. Previous care seeking and testing behavior for HIV is included in the questionnaire as this is primarily an HIV/AIDS survey, but specific questions on care seeking behavior for other STI are not included.*

4. In ethical consideration, please write what study team done to study participants who diagnosed with HIV positive and active syphilis? (referred for available treatment free of cost or with cost etc.).

*Addressed per comment from Reviewer #1.*

5. Write briefly about type of STI services in the country and its coverage in introduction section. Is it free or out of pocket payment etc? Try to link study findings with the availability of STI services in the country in discussion section of the manuscript.

*Addressed in Introduction.*

6. Two-thirds (65.8%) of all participants with syphilis lived in rural areas in country. This is an important finding. Please discuss it in discussion section.

*Thank you for this comment. The significance of this finding is presented in Table 2. This is something the larger PHIA study team has noted and explored for several variables not just syphilis. While true that almost two-thirds of syphilis infections were in rural areas, this result is not significant in our analysis because of the population distribution of Zimbabwe I.e. urban populations are almost entirely limited to Harare and Bulawayo which account for a relatively small percentage of the total population.*

Sincerely,

John H. Rogers, PhD, MPH

CDC PMI Resident Advisor, Nigeria

Formerly:

Associate Director for Science

U.S. Centers for Disease Control and Prevention, Zimbabwe
